# Ribo-seq enlightens codon usage bias

**DOI:** 10.1093/dnares/dsw062

**Published:** 2017-02-07

**Authors:** Damien Paulet, Alexandre David, Eric Rivals

**Affiliations:** 1 Department of Computer Science, Laboratoire d'Informatique, de Robotique et de Microélectronique de Montpellier (LIRMM), CNRS et Université de Montpellier, 34095 Montpellier Cedex 5, France; 2 Institut de Biologie Computationnelle (IBC); Université de Montpellier, France; 3 Cancer Biology Department, Institut de Génomique Fonctionnelle, CNRS, INSERM, Université de Montpellier, F-34094 Montpellier, France

**Keywords:** codon usage, high throughput sequencing, synonymous codon, evolution, translation

## Abstract

Codon usage is biased between lowly and highly expressed genes in a genome-specific manner. This universal bias has been well assessed in some unicellular species, but remains problematic to assess in more complex species. We propose a new method to compute codon usage bias based on genome wide translational data. A new technique based on sequencing of ribosome protected mRNA fragments (Ribo-seq) allowed us to rank genes and compute codon usage bias with high precision for a great variety of species, including mammals. Genes ranking using Ribo-Seq data confirms the influence of the tRNA pool on codon usage bias and shows a decreasing bias in multicellular species. Ribo-Seq analysis also makes possible to detect preferred codons without information on genes function.

## 1. Introduction

Usage of synonymous codons is biased in genomes, as some codons are favoured in highly expressed genes.^1^^,^^2^ This phenomenon, called codon usage bias (CUB), has been observed in most genomes, although it seems to vary according to the complexity of genomes.^1^ The choice of codons seems to depend mainly on tRNA pool: the main hypothesis is that using codons that have the greatest number of accepting tRNAs increases the efficiency or accuracy of translation of highly expressed genes.^3^^,^^4^ In addition to its impact on mRNA translation, CUB has an impact on protein folding.^5^^,^^6^ Despite its importance, precise measures of CUB were, for years, difficult to obtain at genome wide scale. Sharp et al.^7^ developed a clustering approach from a set of sequenced genes: the RSCU for Relative Synonymous Codon Usage. This measure is inferred with a statistical process: two clusters of genes are determined based on expression levels, in order to maximize the difference in RSCU between the two groups, then the RSCU is computed using the codon frequencies within each group. Consequently, RSCU accuracy relies on the number of sequenced genes. Here, we propose a new method that permits to directly compute CUB from all translated genes. A recent technique named ribosome profiling (or Ribo-seq) has revolutionized the analysis of translation and permitted to refine the picture of global gene expression control. Ribo-seq is based on deep sequencing of ribosome-protected mRNA fragments (RPF or “footprints”) and permits genome-wide analysis of translated sequences at nucleotide resolution. From a bioinformatic standpoint, Ribo-seq provides a new type of data: indeed, RPF reveal codon occupancy of active ribosomes.^8^ This technique yields a precise picture of translation by quantifying the number of ribosomes at every position in a genome. Researchers often analyse jointly Ribo-seq data and RNA-seq data and compare the number of mRNAs and the number of mRNAs involved in translation, which allows them to measure the efficiency of translation.^9^^,^^10^^,^^11^^,^^12^^,^^13^ We decided to use only Ribo-seq data to measure CUB, based on a simple rationale: the more observed footprints, the more ribosomes at this position. This means that genes with a high coverage are *highly translated*, and mutations that lead to a benefit in terms of translation are more prone to be fixed in those genes: as a consequence, Ribo-seq could indicate which genes have a strong CUB. Ribo-seq is often used to measure translation efficiency, as the number of ribosomes per copy of an mRNA species. Here *highly translated* (which differs from *efficiently translated*) means that all copies of one mRNA species yield, all together, a large number of proteins.

We ranked genes according to their number of footprints in a precise and limited region of their mRNA (from the 20th to the 200th codons from the start codon). We then split genes into two groups according to their number of footprints and measured codon bias within each group: this way, our two groups are not optimized for RSCU, but derived from translation experiments. Moreover, contrary to previous approaches, we used high-throughput data reflecting the number of ribosomes per sequence and use all translated genes. Therefore, our measure of CUB is directly computed from translational data.

We applied our approach on a variety of genomes from a parasite, like *Plasmodiumfalciparum*, to multicellular eukaryotes like *Caenorhabditiselegans* and *Homosapiens*. We corroborate Sharp’s results on yeast and *C. elegans*,^7,^^14^ but also detect preferred codons in every species. A preferred codon of an amino acid is defined as the codon with the highest frequency in highly expressed genes. Our analysis provides novel insights on the evolution of CUB.

## 2. Materials and methods

### 2.1. ORF selection

Sequences and annotations were collected from NCBI for all organisms. Positions of start codons are crucial in this study. Therefore, we first kept all genes with a unique isoform. Genes with multiple isoforms were rejected if they had different start codons. If all isoforms share the same start codon, we kept the longest common coding sequence. We excluded the mRNAs of mitochondrial genes. Finally, we collected all the spliced mRNA sequences of the selected genes.

### 2.2. Ribo-seq data and mapping

All Ribo-seq data come from already published data (Supplementary Table S1). We chose species belonging to opisthokonts (the Fungi/Metazoa group) for which Ribo-seq data were available and used *P. falciparum* as an outgroup because of its restricted set of tRNA genes compared to all other species considered in this study. When several datasets were available for one species, we selected datasets according to three conditions: 1/availability of a replicate, 2/ribosome profiling that closely follows the protocol of Ingolia et al.,^8^ and 3/a sufficient sequencing depth after quality filtering. For each selected dataset, we plot the footprint position with respect to the start codon for all genes and determined the majority peak near the start. For all experiments, the peak turns out to be at -12 nucleotides upstream from start codon. As explained in the study by Ingolia et al.,^8^ the “shift” applied to the positions of mapped reads was set to 12 nucleotides.

Reads were mapped with CRAC^15^ with a k-mer of length 25: this k-mer is longer than the recommended length for RNA-seq to better fit the short length of RPF. After mapping on mRNA, we took the first position of each read and applied a shift of 12 nucleotides to assign the read to the P-site position of ribosomes.^16^

### 2.3. Categorizing genes into lowly and highly translated genes

For each gene, we computed the coverage by ribosome footprints from the 20th to the 200th codons from the start codon (and at least 20 codons before the stop codon). We computed the mean coverage on the selected region after removal all genes having less than 10 footprints. Then, we ranked genes according to this mean coverage. From this ranking, we create two groups that were made uniform in terms of total amount of footprints: the lowly and highly translated genes. To accentuate the contrast between those two groups, we excluded the top 5% of lowly translated genes and the bottom 5% of highly translated genes.

### 2.4. RSCU computing

Finally, we computed the RSCU as defined by Sharp et al.^7^ within each group (lowly and highly translated genes). Instead of computing the bias of codon usage over the whole ORF, we took sequences from the 20th to the 200th codons from the start codon, i.e. the same region as the one used for gene sorting. For clarity, when the RSCU is computed based on Ribo-seq data, we named it RSCU_RS_. We developed a computer program in Java language to perform this computation (see the Availability section).

### 2.5. Copy number of tRNA genes and clustering

We use the number of tRNA genes of a species as a proxy for the size of its tRNA pool.^17^^,^^18^ We obtained the copy numbers of tRNA genes from the latest version (2.0 from 2016) of the Genomic tRNA Database (GtRNAdb)^19^ for all species, but *Histoplasma capsulatum*. For the latter, we used the Genbank annotation from NCBI database. To cluster species according to their tRNA copy number and according to their average RSCU_RS_, we perform hierarchical clustering with the R software using the Canberra distance with UPGMA algorithm (Unweighted Pair Group Method with Arithmetic Mean). Details are provided in Supplementary Material.

## 3. Results and discussion

Only amino acids that have multiple encoding codons are studied: only 59 codons were used as we excluded ATG (methionine), TGG (tryptophan), and stop codons.

### 3.1. CUB computation

Given a set of mRNA sequences, Sharp has proposed to compute the RSCU for each synonymous codon j of each amino acid i using the formula

$$\frac{n_{i}\times x_{ij}}{\sum_{j=1}^{n_{i}}x_{ij}}$$

where n_i_ denotes the number of synonymous codons for amino acid i, and *x_ij_* denotes the number of occurrences of codon j in the set of sequences. Sharp has selected a subset of mRNAs that were known to be highly expressed. Our proposal is to automatically select mRNA sequences that are highly translated according to their coverage by ribosomes, which is determined by mapping Ribo-seq reads. Then, we use the formula above with counts of codons limited to a range comprised between the 20th and the 200th codons. The choice of this range is explained below.

**Figure 1:**
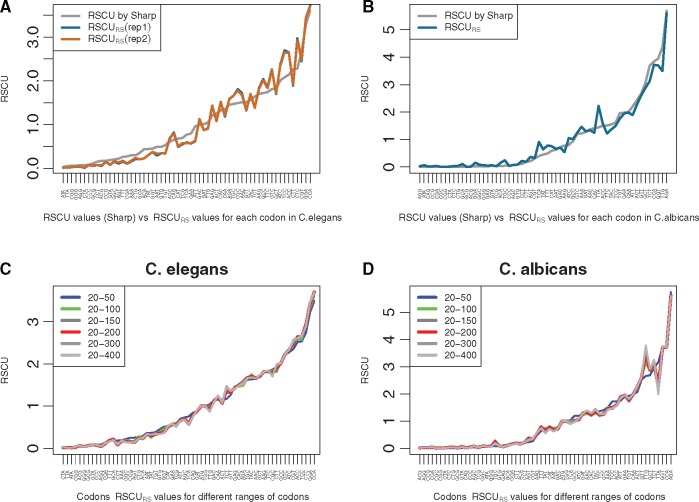
Comparison of the strength of codon usage bias as measured by the RSCU as originally defined by Sharp et al., and by the RSCU_RS_ which is introduced in this work. Both measures are plotted for a subset of highly expressed genes. The RSCU is computed for each codon of an amino acid: it multiplies the relative observed frequency of this codon among all possible codons for the corresponding amino acid by the number of possible codons. In all graphs, the codons on x-axis are ordered by increasing RSCU values computed by Sharp et al. (A) Comparison of RSCU values of Sharp and RSCU_RS_ values in *C. elegans* (in two replicates). Both replicate curves for RSCU_RS_ are extremely close from each other, and they closely follow that from Sharp. (B) Comparison of RSCU values of Sharp and RSCU_RS_ values in *C. albicans*. (C) and (D). Comparison of eight RSCU_RS_ curves obtained with codons counts computed in different ranges of codons starting with 20th codon and ending between the 50th and the 400th codons, respectively, in *C. elegans* (C) and in *C. albicans* (D). Apart for the smallest range of codons (i.e. [20-50]) all curves are very close to each other, thereby showing the robustness of with respect to the range of codons taken into account. One observes that RSCU_RS_ values reach higher values in the yeast species than in the worm species. Refer to the online version for colors.

Sharp and Bradnam^14^ have computed RSCU for *C. elegans*, and we compared his results with our method (RSCU_RS_, for RSCU by Ribo-seq) as illustrated in Fig. 1A. The two methods split genes in two categories: highly and lowly expressed genes, but with completely different inputs: Sharp et al. computed codon bias with a statistical model based on gene sequences, while we used only Ribo-seq footprints. Nevertheless, we obtained a high correlation (>0.97) between Sharp's measure and ours. This correlation is even higher within replicates. In 1992, Lloyd and Sharp^20^ published RSCU values for highly expressed genes in *Candidaalbicans*. Here again, the comparison with RSCU values is shown in Fig. 1B, and we observed a very high correlation between RSCU and RSCU_RS_ (>0.98). In both experiments, we used a larger set of genes (Supplementary Table S1A).

We question whether our method, RSCU_RS_, is robust with respect to the range of codons used—by default [20, 200] codons. The lower limit of 20 avoids counting the accumulation of RPF due to translation initiation.^8^ The upper limit of 200 was chosen as a minimum length for including sufficient counts and to maximize the number of mRNAs taken into account. Clearly, the upper limit choice is somehow arbitrary. We computed the RSCU_RS_ for eight different upper limits ranging from 50 to 400 codons. The RSCU_RS_ curves for each range are shown on a single graph in Fig. 1B for *C. elegans* and Fig. 1C for *C. albicans*. The agreement among all eight curves is striking for both species. Only the curves of two shortest ranges—[20-50] and [20,100] —depart slightly from the other curves, indicating that a minimum number of codons is necessary to capture a stable signal. All other curves are very close to each other, showing that the RSCU_RS_ results are not strongly dependent of the upper limit. Hence, RSCU_RS_ appears to be robust with respect to this parameter. Since the curve for range [20,200] is highly correlated with Sharp's curves, logically all eight curves are highly correlated with Sharp's RSCU values as well.

### 3.2. CUB exists in all species

**Figure 2:**
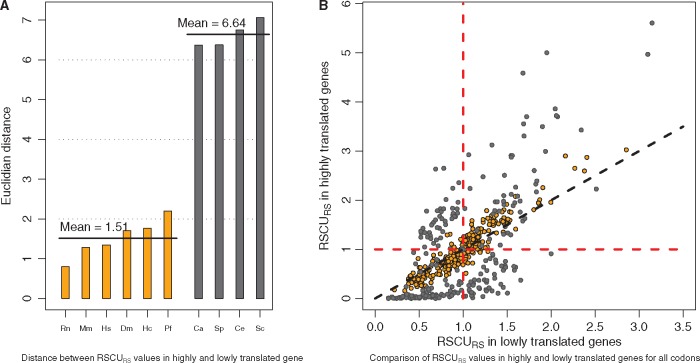
Differences in codon usage bias between lowly and highly translated genes. (A) Euclidean distance between RSCU_RS_ of lowly and highly translated genes was computed for ten species. This distance clearly partitions the species in two groups: the mean euclidean distance is 1.51 for group 1 (left, in orange), and is 6.64 for group 2 (right, in grey). Group 2 comprises *C. albicans, S. cerevisiae, S. pombe* and *C. elegans*, while group 1 comprises all vertebrates, *D. melanogaster, P. falciparum* and *H. capsulatum*. (B) For each species, comparison of the RSCU_RS_ of all codons between lowly and highly translated genes (ltg vs htg). Codons from group 1 species (containing the vertebrates) are shown as orange circles, while codons from group 2 species (that of the budding yeast) as gray circles. A point near the diagonal indicates a similar behavior in ltg and htg. The further apart the point from the diagonal, the higher the bias of that codon. Refer to the online version for colors.

As a first step, we computed the Euclidean distance of RSCU_RS_ between highly and lowly translated genes (Fig. 2A) as a mean to evaluate the intensity of CUB for each organism: if this distance is close to 0, it means there are very few differences in codon usage between highly and lowly translated genes. Results gave two clearly distinct species groups. The first group corresponds to species with a lower CUB and comprises mammals, Drosophila, *P. falciparum*, and *H. capsulatum*. The second group of species exhibiting a higher CUB contains three yeasts and *C. elegans*.

For these species, we compare the RSCU_RS_ of all codons between lowly and highly translated genes (ltg vs htg; Fig. 2B). One notices the asymmetry of the diagram with respect to the diagonal: The part of the diagram beyond a RSCU_RS_ of two is populated above the diagonal and almost empty below it. This confirms the existence of favoured codons in htg, and their absence in ltg. Codons located far above the diagonal indicate strongly favoured codons in htg. This illustrates the impact of selection pressure linked to the gene expression level. There also exist codons far below the diagonal (which are disfavoured in htg), but their RSCU_RS_ remains low (<2): those are either disfavoured or slightly favoured in ltg. Some codons even exhibit a nearly null RSCU_RS_ in htg, meaning these are almost forbidden in htg. Apart from the global asymmetry, the difference between the codons of the two species groups is striking. All codons far apart from the diagonal belong to group 2 and very few codons of these species lie near the diagonal: in a species like yeast, the difference of usage between htg and ltg is strong for most codons. Note also that in group 2, some codons are disfavoured in htg and favoured in ltg, suggesting there could be some selection pressure also in ltg, albeit weaker than in htg. In species of group 1, most codons lie around the diagonal, but the bias changes with the RSCU_RS_ value: most codons that are disfavoured in htg (dots lying below the horizontal line, i.e. with a RSCU_RS_ in htg <1) lie below the diagonal, while most codons favoured in htg (i.e. a RSCU_RS_ in htg > 1) lie above the diagonal. This suggests that codon usage preference in htg is small, but is detectable with Ribo-seq data, and thus do exist also in these species.

### 3.3. Patterns of codon preferences

**Figure 3:**
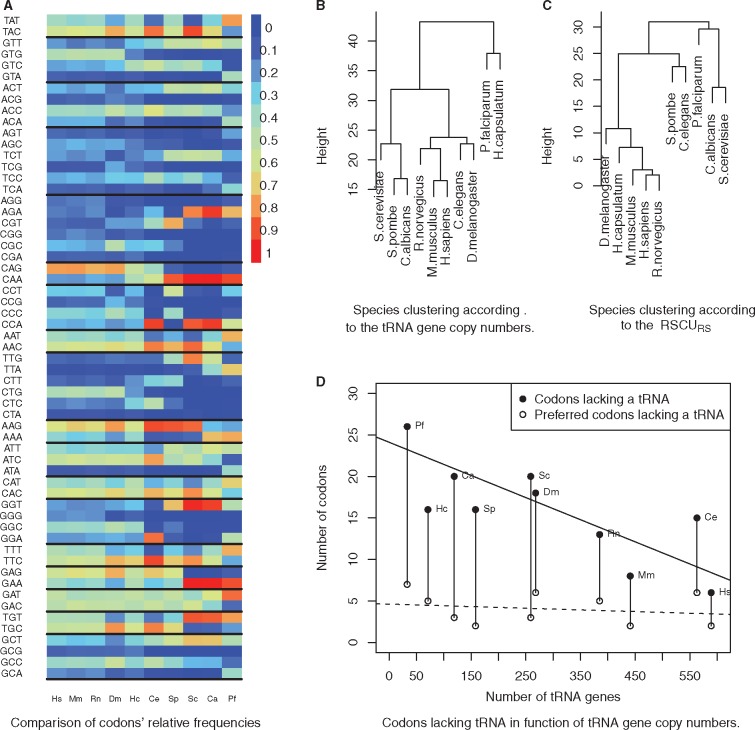
(A) Preferred codons in highly translated genes. To ease the comparison of the bias across codons, we plotted the codon frequency such that each codon has a value comprised in [0,1]. The frequency is the RSCU multiplied by the number of codons for the corresponding amino acid. The range of colors goes from blue for a frequency of 0 until red for a frequency of 1. Same species abbreviations as in Fig. 1. (B) Clustering of the ten species based on tRNA copy numbers. (C) Clustering of the 10 species based on RSCU_RS_ in highly translated genes. Both clustering were performed using UPGMA algorithm in R (see Methods section). (D) Variation of the numbers of codons lacking tRNA with the size of the species' tRNA pool. Linear regression lines are solid for codons with no tRNA (*R*^2^ = 0.69) and dotted for preferred codons with no tRNA (*R*^2^ = 0.53). *H. capsulatum* was excluded for both regressions. The regression line for all codons decreases sharply (slope = −0.026), while the regression line for preferred codons remains flat (slope = −0.002). Refer to the online version for colors.

Then, we compared the frequency of codons in highly translated genes. Higher eukaryotes have few strongly preferred codons while yeasts show marked differences (Fig. 3A): some yeasts present a highly preferred codon for Arginine, Glutamic acid and Cysteine, whereas mammals and Drosophila do not. Glutamine follows remarkable patterns: both mammals and Drosophila strongly prefer CAG, yeasts favour CAA, while *H. capsulatum* and *C. elegans* use CAA/CAG in an equivalent fashion.

Our analysis revealed that some preferred codons are well conserved (Table 1): all species, but *P. falciparum*, have the same preferred codon for phenylalanine, histidine, tyrosine (that is, all aromatic amino acids that can be studied), and asparagine. On the opposite, Leucine has five different preferred codons. Indeed, unique preferred codons are rare: *Schizosaccharomycespombe* has one only for proline (CCT), and *P. falciparum* has the highest number of unique features with seven unique preferred codons.

**Table 1:** Preferred codons per organism in highly (A) and lowly (B) translated genes

A.										
	Hs	Mm	Rn	Dm	Hc	Ce	Sp	Sc	Ca	Pf
Phe	TTC	TTC	TTC	TTC	TTC	TTC	TTC	TTC	TTC	TTT*
His	CAC	CAC	CAC	CAC	CAC	CAC	CAC	CAC	CAC	CAT
Tyr	TAC	TAC	TAC	TAC	TAC	TAC	TAC	TAC	TAC	TAT*
Asn	AAC	AAC	AAC	AAC	AAC	AAC	AAC	AAC	AAC	AAT*
Lys	AAG	AAG	AAG	AAG	AAG	AAG	AAG	AAG	AAA	AAA
Cys	TGC	TGC	TGC	TGC	TGC	TGC	TGC	TGT*	TGT*	TGT
Glu	GAG	GAG	GAG	GAG	GAG	GAG	GAG	GAA	GAA	GAA
Ala	GCC*	GCC*	GCC*	GCC*	GCC*	GCC*	GCT	GCT	GCT	GCT
Se	TCC*	TCC*	TCC*	TCC*	TCC*	TCC*	TCT	TCT	TCT	TCA
Thr	ACC*	ACC*	ACC*	ACC*	ACC*	ACC*	ACT	ACT	ACT	ACA
Ile	ATC*	ATC*	ATC*	ATC*	ATC*	ATC*	ATT	ATC*	ATT	ATT
Gly	GGC	GGC	GGC	GGC	GGC*	GGA*	GGT*	GGT*	GGT*	GGA
Pro	CCC	CCC*	CCC*	CCC*	CCC*	CCA	CCT	CCA	CCA	CCA
Gln	CAG*	CAG*	CAG*	CAG*	CAG*	CAA	CAA	CAA	CAA	CAA
Leu	CTG*	CTG	CTG	CTG*	CTC*	CTC*	TTG*	TTG	TTG	TTA
Val	GTG	GTG	GTG*	GTG	GTC*	GTC*	GTT	GTT	GTT	GTT
Arg	CGC*	CGC*	CGC*	CGC*	CGC*	CGT	CGT	AGA	AGA	AGA
Asp	GAT*	GAC	GAC	GAC	GAT*	GAC	GAT*	GAC	GAT*	GAT*

B.										
	Hs	Ms	Rn	Dm	Hc	Ce	Sp	Sc	Ca	Pf
Phe	TTT	**TTC**	**TTC**	**TTC**	**TTC**	**TTC**	TTT	TTT	TTT	**TTT**
His	**CAC**	**CAC**	**CAC**	**CAC**	CAT	CAT	CAT	CAT	CAT	**CAT**
Tyr	**TAC**	**TAC**	**TAC**	**TAC**	**TAC**	TAT	TAT	TAT	TAT	**TAT**
Asn	AAT	**AAC**	**AAC**	**AAC**	AAT	AAT	AAT	AAT	AAT	**AAT**
Lys	**AAG**	**AAG**	**AAG**	**AAG**	**AAG**	AAA	AAA	AAA	**AAA**	**AAA**
Cys	**TGC**	**TGC**	**TGC**	**TGC**	**TGC**	TGT	TGT	**TGT**	**TGT**	**TGT**
Glu	**GAG**	**GAG**	**GAG**	**GAG**	GAA	GAA	GAA	**GAA**	**GAA**	**GAA**
Ala	**GCC**	**GCC**	**GCC**	**GCC**	**GCC**	GCT	**GCT**	**GCT**	**GCT**	**GCT**
Ser	AGC	AGC	AGC	AGC	TCC	TCA	**TCT**	**TCT**	TCA	AGT
Thr	**ACC**	**ACC**	**ACC**	**ACC**	**ACC**	ACA	**ACT**	**ACT**	**ACT**	**ACA**
Ile	**ATC**	**ATC**	**ATC**	**ATC**	**ATC**	ATT	**ATT**	ATT	**ATT**	ATA
Gly	**GGC**	**GGC**	**GGC**	**GGC**	**GGC**	**GGA**	**GGT**	**GGT**	**GGT**	**GGA**
Pro	**CCC**	**CCC**	**CCC**	**CCC**	CCA	**CCA**	**CCT**	**CCA**	**CCA**	**CCA**
Gln	**CAG**	**CAG**	**CAG**	**CAG**	**CAG**	**CAA**	**CAA**	**CAA**	**CAA**	**CAA**
Leu	**CTG**	**CTG**	**CTG**	**CTG**	**CTC**	CTT	TTA	**TTG**	TTA	TTA
Val	**GTG**	**GTG**	**GTG**	**GTG**	**GTC**	GTT	**GTT**	**GTT**	**GTT**	GTA
Arg	**CGG**	**CGG**	**CGG**	**CGG**	**CGG**	AGA	**CGT**	**AGA**	**AGA**	**AGA**
Asp	**GAT**	**GAC**	**GAC**	**GAC**	**GAT**	GAT	**GAT**	GAT	**GAT**	**GAT**

Color code: on a blue background codons shared with *M. musculus*, on a red background, codons shared with *C. albicans.* A: Bolded codons with a star are preferred codons for which a codon with more acceptors exists. B: bolded and underlined codons are identical in lowly and highly translated genes. The following abbreviations of species names are used throughout this article: H. sapiens (Hs), *H. sapiens (Hs)*, *M. musculus (Mm)*, *R. norvegicus (Rn)*, *D. melanogaster (Dm)*, *C. elegans (Ce), H. capsulatum (Hc), S. pombe (Sp)*, *S. cerevisiae (Sc), C. albicans (Ca*), and *P. falciparum (Pf)*. Refer to the online version for colors.

### 3.4. Preferred codons and tRNA copy number

It is assumed that a codon with the highest copy number of corresponding tRNAs is prone to be the preferred codon. This result is confirmed in all species, but *H. capsulatum* (Table 2). Nevertheless, species do not equally observe this tendency: in *S. pombe* 15 out 18 amino acids prefer the codon with the highest copy number of tRNA, while only 9-12 amino acids behave the same in mammals. Non-majority preferred codons are shared among species, like species preferring GGT for glycine (*S. pombe*, *Saccharomycescerevisiae* and *C.albicans*) or preferring GCC for Alanine (*H. sapiens*, *Musmusculus*, *Rattusnorvegicus*, *Drosophilamelanogaster*, *C. elegans* and *H. capsulatum*).

**Table 2:** Comparison of favourite codons and tRNA copy numbers

Species	Number of tRNAs	Number of favourite codons having the highest tRNA copy number (out of 18 cases)	(%)	Number of favorite codons lacking tRNA	Number of codons lacking tRNA
*H. sapiens*	589	9	50.0	2	6
*C. elegans*	563	12	66.7	6	15
*M. musculus*	441	12	66.7	2	8
*R. norvegicus*	385	10	55.6	5	13
*D. melanogaster*	268	12	66.7	6	18
*S. cerevisiae*	259	15	83.3	3	20
*S. pombe*	158	15	83.3	2	16
*C. albicans*	119	15	83.3	3	20
*H. capsulatum*	71	8	44.4	5	16
*P. falciparum*	33	14	77.8	7	26

(The codons for Stop, Met and Trp are excluded). tRNA: transfer RNA. Copy numbers of tRNA genes come from GtRNAdb 2.0^19^. The percentage of favourite codons (one per amino acid) that have the highest tRNA copy number lies above 50% for all species but *H. capsulatum*.

We inferred two species trees based on the species' proximity either in terms of tRNA copy number, or in terms of average RSCU_RS_ over highly translated genes (Fig. 3B and C). These trees are similar, but not equal. tRNA copy number groups the mammals with Drosophila and *S. cerevisiae*, and makes a clade with *C. albicans*, *S. pombe* and *C. elegans*, while both *H. capsulatum and P. falciparum* form an outgroup (which reflects their small tRNA gene copy numbers; see Table 2). With CUB (Fig. 3C), the clade grouping the mammals and Drosophila remains opposed to the branches containing the yeast species and *P. falciparum*. The striking feature is the position of *H. capsulatum*, which, due to its low tRNA gene number, is located at the base the tRNA-tree, but it is grouped with the mammals in the CUB-tree. Despite its basal position in the tree, *H. capsulatum* is closer to the group of yeasts (Euclidian distance = 29.9) than to the group of mammals (Euclidian distance = 86.5).

### 3.5. Preferred codons, codons lacking tRNA and GC content

Wobbling corresponds to a codon that is decoded without following Watson–Crick base pair rules.^21^ Wobbling is complex to study, but codons lacking tRNA are necessarily subject to it. In Fig. 3D, we compared the numbers of codons lacking tRNA, and of preferred codons lacking tRNA for all species ordered by the size of their tRNA pool. As expected, when the tRNA pool increases, the number of codons lacking tRNA decreases steeply (slope equals −0.026642). However, the number of preferred codons lacking tRNA remains stable and appears not to depend on the size of the tRNA pool (slope dashed curve equals −0.002812).

**Figure 4:**
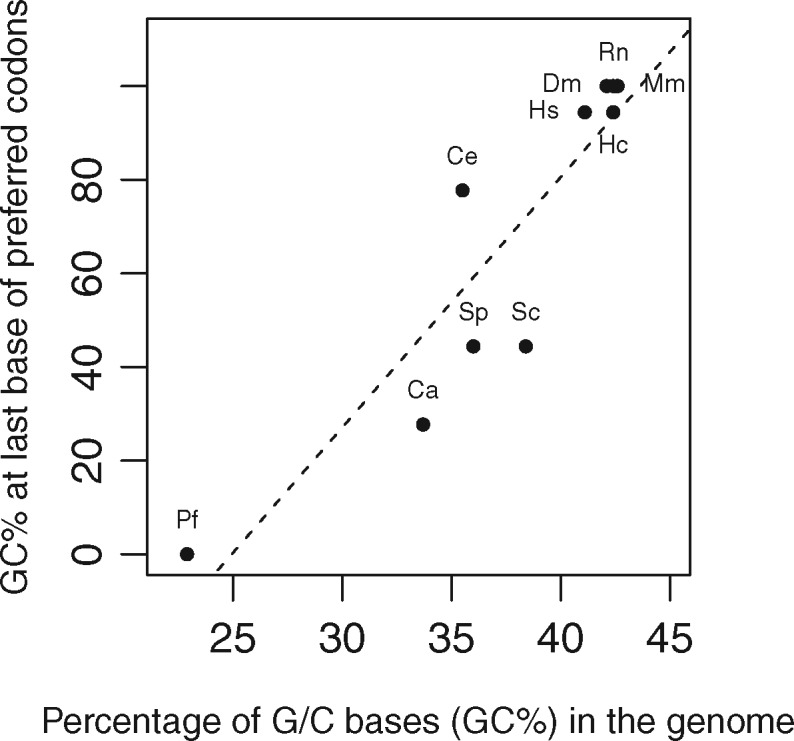
Genome percentage of GC bases (GC %) versus GC % of last base of preferred codons. Same species abbreviations as in Fig. 1.

The last base of a codon is supposed to correlate with the GC content of a genome. This result is partially confirmed in Fig. 4. On one extremity, *P. falciparum* has a very low GC content and no preferred codon ending with a G or C. At the other extremity, all species with a high GC content strongly prefer codons ending with a G or C. For intermediate values of GC content, results are more puzzling: *C. elegans* has an intermediate GC content compared to *C. albicans*, *S. pombe* and *S. cerevisiae*, but has a much stronger preference for codons ending with a G or C.

## 4. Conclusion and discussion

CUB measures the difference in the frequency of translation of each possible codon for an amino acid. CUB is detected in highly expressed genes because the selection pressure to choose optimal codons is stronger in those genes. First, it is known that numerous mRNAs are finally not translated^22^ and have thus no impact on fitness. Hence, to measure CUB, Ribo-seq seems more appropriate to select highly expressed genes than RNA-seq data. Second, various forces and processes (such as mutation bias) impact the nucleotidic composition of a gene.^17^ Because it captures the presence of ribosomes, Ribo-seq allows to measure the sole translational advantage of a codon rather than the impact of evolutionary forces unrelated to translation.

Our method based on Ribo-seq—rather than RNA-seq—data allows us to compute a precise and direct measure of CUB. Although Ribo-seq was not originally designed for CUB studies, we show that our method assesses the CUB with high precision. Using translation data provides other benefits: first, all translated genes can be included even if functional information is lacking. Second, our method avoids any statistical optimization as CUB is directly computed from translation data. Third, Ribo-seq data allows to compute CUB and to detect preferred codons in a wide variety of species. Fourth, our method may help to measure variation of CUB across conditions or tissues. Nevertheless, the number of Ribo-seq footprints per gene depends on the sequencing depth, which may introduce some bias if depth is low. To correctly classify genes, this method also requires a good sequencing depth in order to get at least some footprints on lowly translated genes: for example, we can grossely estimate that it requires at least 3,000 covered genes to study human's CUB.

Our method was validated by comparison to previous results and our results confirm the current knowledge on CUB. First, the CUB is stronger in highly translated genes.^7^ Second, most higher eukaryotes exhibit a weaker CUB when compared to unicellular species, like yeasts.^17^ Third, tRNA copy number has a strong influence on preferred codons, as they indicate preferred codons for most of amino acids.^18^ Nevertheless, the tRNA copy number is not the only criterion: the GC content also influences preferred codons, but to a lesser extent than the tRNA pool. The clustering of pluricellular species in Fig. 3B and C suggests that translation can adapt to a changing GC content. Surprisingly, an increasing tRNA pool size does not diminish the number of preferred codons lacking tRNA.

Ribo-seq data on a larger set of species would help to get a better understanding of CUB evolution, but to date, only very few species have been subjected to Ribo-seq experiments. Nevertheless, comparison of codon bias between species seems to reveal a common story that is partially adapted by every organism. In yeast, the level of ribosome occupancy measured using an adequate protocol was found to be negatively correlated with tRNA copy numbers, which is used as a proxy of cognate tRNA abundances.^13^ Our results suggest that the tRNA pool is the key factor for every amino acid in a wide range of species, and that some preferred codons are conserved across the tree of life. However, we clearly distinguished two species groups when comparing the intensities of CUB. The low-intensity group contains mammals and drosophila (plus *P. falciparum* that has a very restricted tRNA pool, which may be due to the difficulty of tRNA gene prediction, and *H. capsulatum* which we discussed later) versus a three-time higher CUB intensity group formed by *C. elegans*, *S. pombe*, *C. albicans* and *S. cerevisiae*. Surprisingly, *C. elegans* does not belong to the low intensity group, while mammals and drosophila do. It has common features with both groups: some amino acids with a yeast-like preferences (like valine or proline), some with a mammals-like preferences (like glutamic acid or aspartic acid) and some with a unique profile (glycine and leucine). A possible scenario could be a decreasing intensity of CUB in multicellular species: nonetheless, this evolution would be achieved progressively, amino acid by amino acid (often through a substitution at the third base of a codon^23^—see examples below). Highly translated genes are more subjected to CUB than lowly translated genes and, as shown in Table 1, a general pattern appears from yeasts to mammals: an evolution CAT to CAC for Histidine, from GCT to GCC in Alanine, for example. This general pattern admits some exceptions: for instance, *H. capsulatum* and *C. elegans* favour GTC for valine whereas the common pattern favours an evolution from GTT to GTG. Besides the general tendencies, CUB evolution is to some degree dependent of the species, as suggested by some species specific preferred codons. Including more species would help to further unravel the evolution of CUB, or to investigate the generality of its impacts that were detected only in some species^24^^,^^25^; thus we hope that Ribosome profiling will be applied to an increasing number of species.

The most striking results were obtained for the pathogenic yeast, *H. capsulatum*. Its tRNA pool is closer to that of other yeasts, but its CUB much closer to that of mammals. This suggests that its CUB is quite independent of its tRNA pool. As *H. capsulatum* is a pathogen of mammals, it is tempting to say that it adapts to its host, but we lack information to conclude. Is *H. capsulatum* able to use the translational machinery of its host (ribosomes and/or tRNAs) or do more tRNA genes remain to be annotated in its genome? Including other pathogenic species in the comparison, such as the fungi Aspergillus, would help to determine whether this feature is specific to *H. capsulatum* or widespread in pathogens. Nevertheless, pathogens could represent an exception in which the CUB depends more on the host's tRNA pool than on their own tRNA pool. 

## Availability

The program for computing the codon usage bias from Ribo-seq is freely available at: http://www.lirmm.fr/∼rivals/rscu

## Supplementary Material

Supplementary Data
