# Supplementary material for: Ribo-seq enlightens codon usage bias
Source: DNA Res. 2017 Feb 7;24(3):303–210. doi: 10.1093/dnares/dsw062 (PMC5499818; doi:10.1093/dnares/dsw062)
Supplement: Supplementary Data [file dsw062_Supp.pdf]

# Ribo-seq enlightens Codon Usage Bias

# Supplementary material

A.

| | Shrap | This study |
|---|---|---|
| C. elegans | 178 | 181 |
| C. albicans | 28 | 113 |

B.

| Organism | Sample | Experiment |
|---|---|---|
| M. musculus | SAMN03098049 | SRR1605293 |
| H. sapiens | SAMN00990790 | SRR493747 |
| D. melanogaster | SAMN02264622 | SRR942868 |
| H. capsulatum | SAMN03649616 | SRR2015214 |
| P. falciparum | SAMN02850772 | SRR1378560 |
| R. norvegicus | SAMN03008628 | SRR1557705 |
| C. elegans | SAMN01090422 | SRR522871 |
| | | SRR522872 |
| S. pombe | SAMEA2292866 | ERR392085 |
| C. albicans | SAMN02400884 | SRR1027795 |
| S. cerevisiae | SAMN00002142 | SRR014378 |

Table 1.

A. Number of genes used in highly expressed genes in Sharp and this study.

B. Species and Ribo-seq samples used in this study.

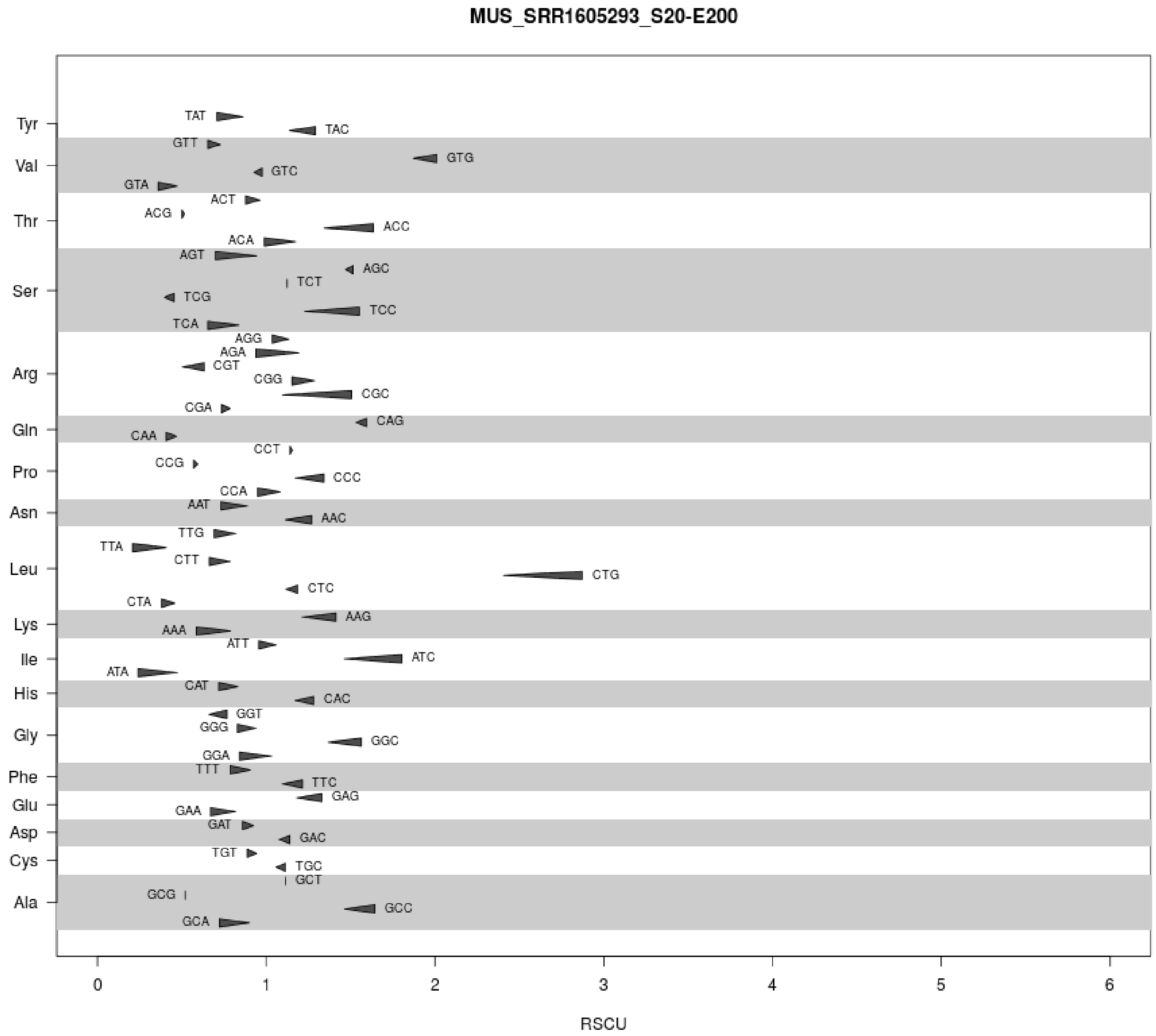


Figure 1. RSCU$_{RS}$ in *M. musculus*. Large borders of triangles represent RSCU$_{RS}$ in highly expressed genes, and peaks represent RSCU$_{RS}$ in lowly expressed genes.

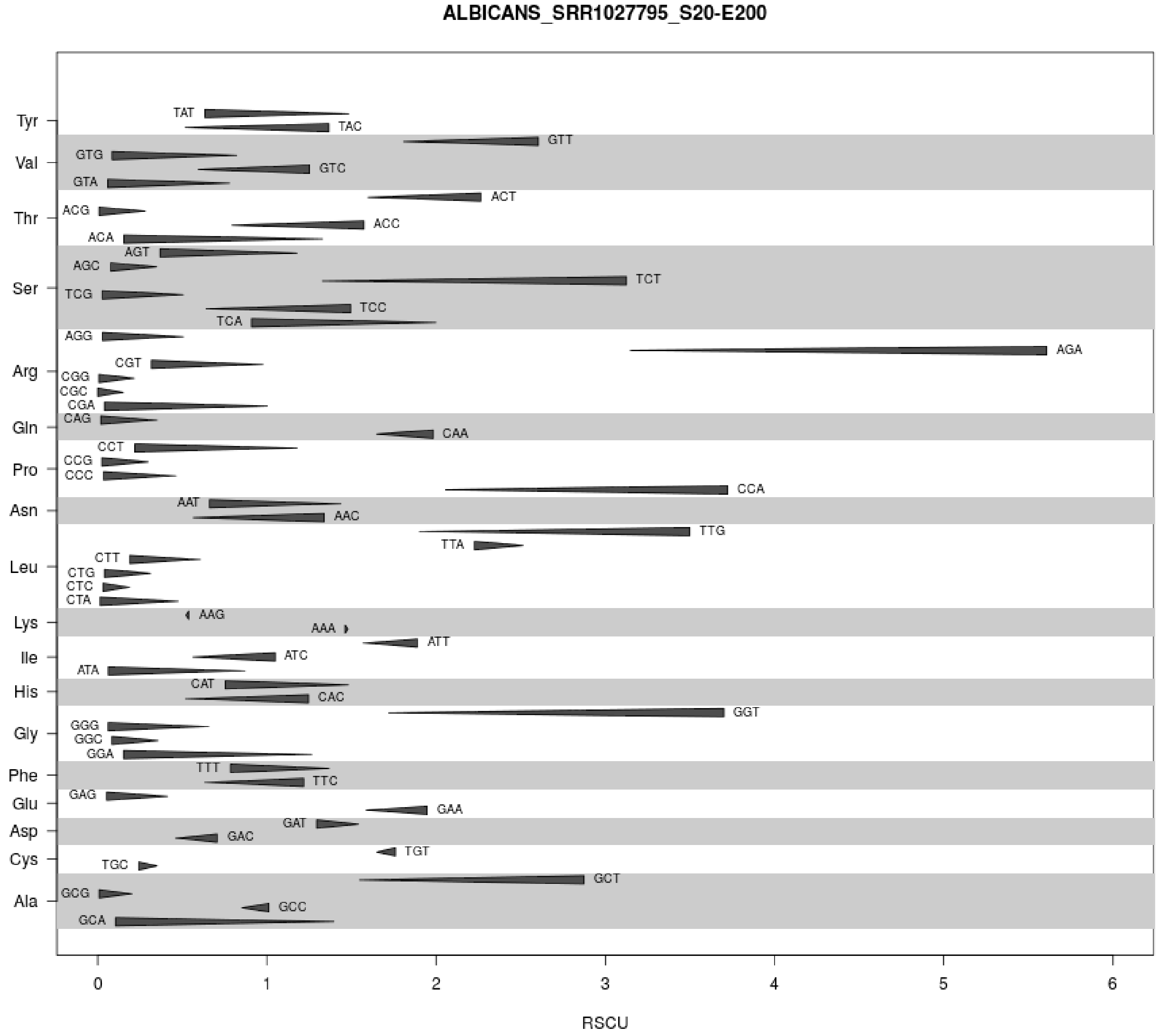


Figure 2. RSCU$_{RS}$ in *C. albicans*. Large borders of triangles represent RSCU$_{RS}$ in highly expressed genes, and peaks represent RSCU$_{RS}$ in lowly expressed genes.

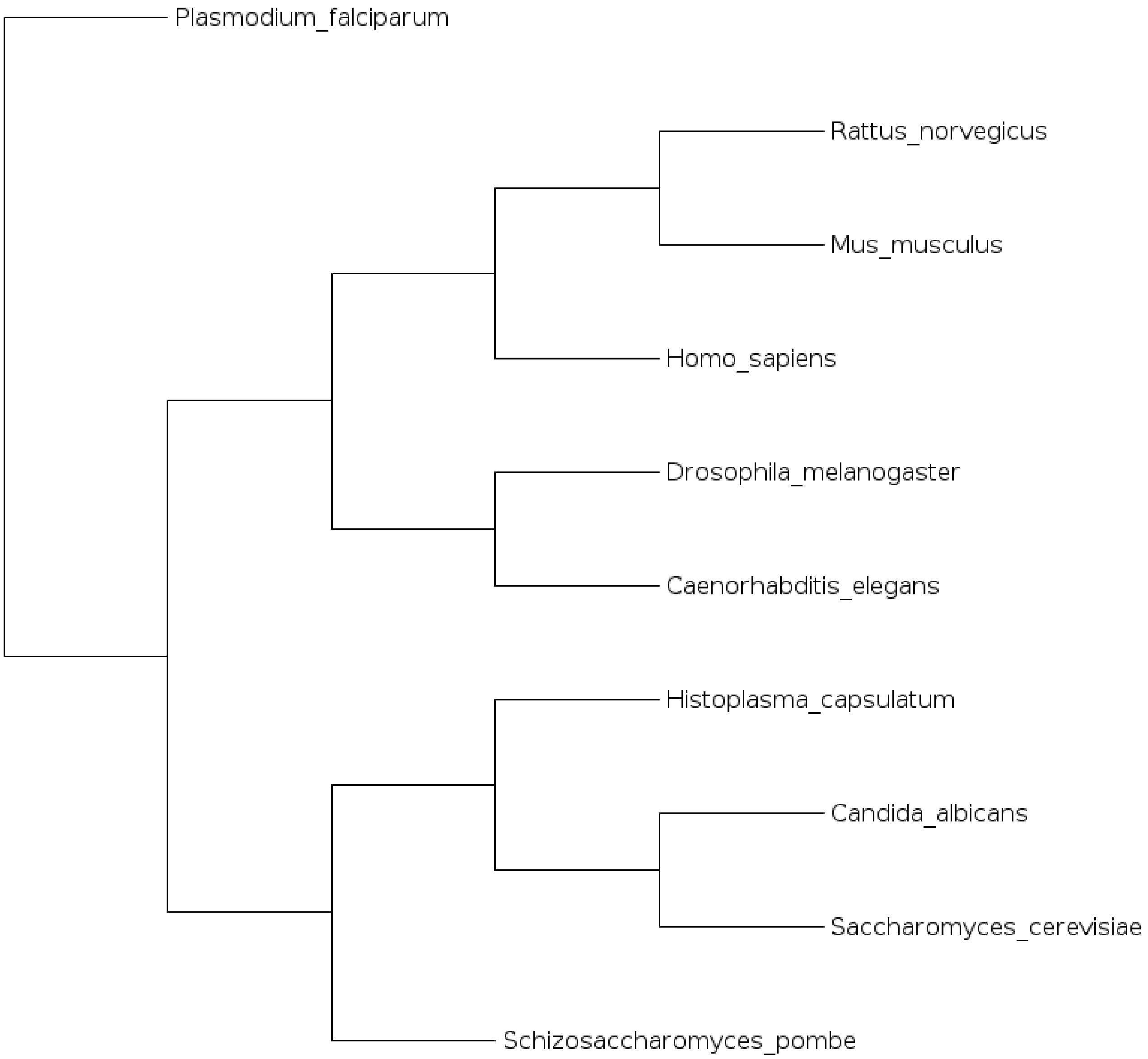

Figure 3. Species tree taken from the NCBI taxonomy.
